# Numerical study on the stimulation effect of boundary sealing and hot water injection in marine challenging gas hydrate extraction

**DOI:** 10.1038/s41598-024-66321-5

**Published:** 2024-07-03

**Authors:** Shuaishuai Nie, Ke Liu, Kangtai Xu, Xiuping Zhong, Shixing Tang, Jian Song, Hongjing Zhang, Jiangfei Li, Yafei Wang

**Affiliations:** 1 School of Petroleum Engineering, Hebei Petroleum University of Technology, Chengde, 067000 China; 2 College of Construction Engineering, Jilin University, Changchun, 130026 China; 3 Sulige Gas Field Development Company, PetroChina Changqing Oilfield, Xi’an, 710018 China

**Keywords:** Gas hydrate, Open boundary, Reservoir stimulation, Methane leakage, Water control, Energy science and technology, Fossil fuels

## Abstract

This study proposed a novel development mode combining boundary sealing and hot water injection to address the challenges of gas leakage, limited reservoir sensible heat, boundary water intrusion, and low productivity faced by challenging hydrate extraction, and the stimulation effect was numerically investigated with Shenhu hydrates as the geological background. The results showed that lower boundary permeability facilitated pressure propagation and achieved volumetric dissociation of hydrates, whereas insufficient formation energy resulted in substantial gas retention. Hot water injection was effective for stimulation, but open boundaries could not maintain the high injection pressure, leading to massive hot water losses and gas escapes. However, their combination achieved a synergistic stimulation like “1 + 1 > 2” because a piston water drive similar to secondary recovery in oil and gas development was formed. Relative to three-spot well patterns, the five-spot shortened the extraction cycle by 680 days and enhanced the gas-to-water ratio by 17%. Increasing injection pressure enhanced water yield more significantly while the improvement of gas yield was more significant by increasing hot water temperature. Overall, high-pressure and high-temperature injection was suggested for gas enhancement and water control. These findings provide important guidance for advancing the commercial development of challenging hydrates.

## Introduction

Natural gas hydrate (NGH) is a new energy source widely distributed on submarine slopes and permafrost zones, where gas molecules are encased in polyhedral cages constructed from water molecules connected by hydrogen bonds^1^. Due to its huge reserves, high energy density, and clean combustion, is considered a promising potential energy resource that plays a vital role in the future climate environment^2^.

Since the 1980s, a worldwide wave of research on NGH resource investigation and development technologies has arisen. So far, more than 230 NGH-rich areas have been discovered in 79 countries, and approximately 97% of the global NGH resources are deposited in submarine sediments^3^. Due to the complex marine environments, geological conditions, and phase behavior, NGH development remains at the exploratory stage, with only four marine NGH production trials conducted in the Nankai trough of Japan^4^ and Shenhu Area of the South China Sea^5^. In contrast to the sandy deposits in the Nankai trough, Shenhu hydrates are hosted in clayey-silty sediments with low reservoir permeability and permeable boundary layers^6^. Such host environments contain more than 90% of the global NGH resources and are known as challenging hydrates^7^. This challenge is twofold: first, the low reservoir permeability significantly increases the difficulty of NGH dissociation and gas recovery^2,7–10^, and second, the permeable boundary layers exacerbate the risk of methane leakage^11–16^. As a result, searching for effective and reliable extraction methods has been a pressing issue.

The general idea of NGH extraction is to break its phase equilibrium state in situ and subsequently recover the decomposed gas, with depressurization, inhibitor injection, thermal stimulation, and gas replacement as primary methods. Of these, depressurization is generally considered the most promising because of its relative ease of implementation, with productivity controlled by pressure relief potential as well as heat and mass transfer^17^. However, for challenging hydrates, pressure propagation, fluid flow, and enthalpy available are suppressed by the low reservoir permeability and open boundaries. Su et al. (2012) found that lowering the permeability of boundary layers promoted pressure drop propagation and that the increased gas volume improved the gas-phase relative permeability, resulting in higher productivity^10^. Sun et al. (2015) pointed out that permeable underburden was superior to that of permeable overburden as warmer bottom water intrusion favored heat supply^18^. Bhade and Phirani (2015) found that the NGH reservoir with an unconfined aquifer resulted in ineffective depressurization^19^. Li et al. (2021) concluded that the completion section should contain the underburden at a reservoir permeability of 1–100 mD^20^. Ning et al. (2022) found that reducing the permeability of boundary layers strengthened the synergistic pressure-relief effect of multi-branch wells and increased long-term gas production^21^. The above-mentioned studies indicate that the boundary effect can not be ignored in challenging hydrate extraction, that is, permeable boundary layers are conducive to the environmental enthalpy available, but also weaken pressure propagation and result in high water production, a bottom-water intrusion phenomenon that should be avoided in conventional gas reservoirs. As a result, challenging hydrate extraction by depressurization alone makes it hard to reach the desired production performance.

The combination of depressurization and thermal stimulation has been demonstrated to be a more efficient extraction method. The heating means include fluid-free methods such as electricity and magnetism, as well as fluid-containing methods such as thermal fluid injection. The heating range of fluid-free methods is limited due to the low thermal conductivity of sediments, whereas the fluid-containing methods with convective heat transfer are considered more efficient^22^. Particularly, injection-production well patterns such as two-, three-, and five-spot exhibit tantalizing productivity and have been proposed for commercial development by Japan^23,24^. This method may be applicable to sandy NGH reservoirs; however, for challenging hydrates, the decomposed gas and injected fluid may migrate to the boundary layers driven by the high injection pressure, resulting in ineffective thermal stimulation and greatly increasing the risk of methane leakage. Numerical investigation on Shenhu hydrates showed that the gas escaping under a unit horizontal well section reached 15080 m^3^ at a well spacing of 100 m^16^. In our previous studies, the escaped gas was successfully recovered by placing two capture wells at the boundary layers. However, due to the small spacing between the capture and injection wells, premature water flooding greatly weakened the thermal stimulation effect and resulted in high water yield^16,25^. Consequently, an urgent issue is how to enhance gas productivity while controlling water and avoiding gas leakage.

Water plugging is an effective technology for water control in the development of oil and gas reservoirs. The general technology idea is to block the water-bearing layer or water flow channel with sealing and plugging materials such as cement, gels, polymers, fibers, and Fuzzy-ball, to reduce the water-phase relative permeability^26–28^. In the case of challenging hydrates, the open boundary conditions are similar to those of oil and gas reservoirs containing edge or bottom water. A viable option is to create artificial barriers by injecting sealing and plugging materials at the boundaries, thus cutting off the hydraulic connection between the reservoir and aquifer^29–32^. Furthermore, the low-permeability sealing zones could increase the flow resistance of decomposed gas and hot water to the boundary layers, which may be a promising technology for the safe and efficient development of challenging hydrates.

This study proposed a novel extraction method combining boundary sealing and hot water injection to address issues such as CH_4_ leakage, boundary water intrusion, insufficient reservoir sensible heat, as well as poor gas production in challenging hydrate development, and the stimulation potential of boundary sealing or/and hot water injection was numerically evaluated, with Shenhu hydrates at the SH2 site as the research object. Furthermore, the effects of key factors including well pattern types, boundary permeability (k_b_), injection pressure (P_i_), and hot water temperature (T_i_) on hydrate decomposition and gas–water production were investigated. The findings of this study could provide important guidance for the commercial development of challenging hydrates.

## Numerical modeling

### Geological background

Shenhu Area, located in the northern land slope of the South China Sea, with a seawater depth of 900–1500 m, is one of the most active areas for marine hydrate exploration and exploitation. Since 2007, three cruises have been conducted, and numerous hydrate samples have been recovered, with hydrate saturation (S_h_) of 20–48% and CH_4_ content exceeding 99.7%^6,33–35^. Among them, the NGH reservoir at the SH2 site has the highest S_h_ value of 47.3% and a maximum reservoir thickness of 43 m, and thus has development potential and represents the typical storage characteristics of Shenhu hydrates^36^. The core analysis further revealed that the permeabilities of the reservoir-cover sediment were 10 mD, and consisted of less than 2% sand, 60–80% silt, and 15–45% clay^37,38^. As a result, challenging hydrates at the SH2 site were taken as the research object to explore the stimulation effect of boundary sealing and hot water injection.

### Development plans and simulation scheme

**Figure 1:**
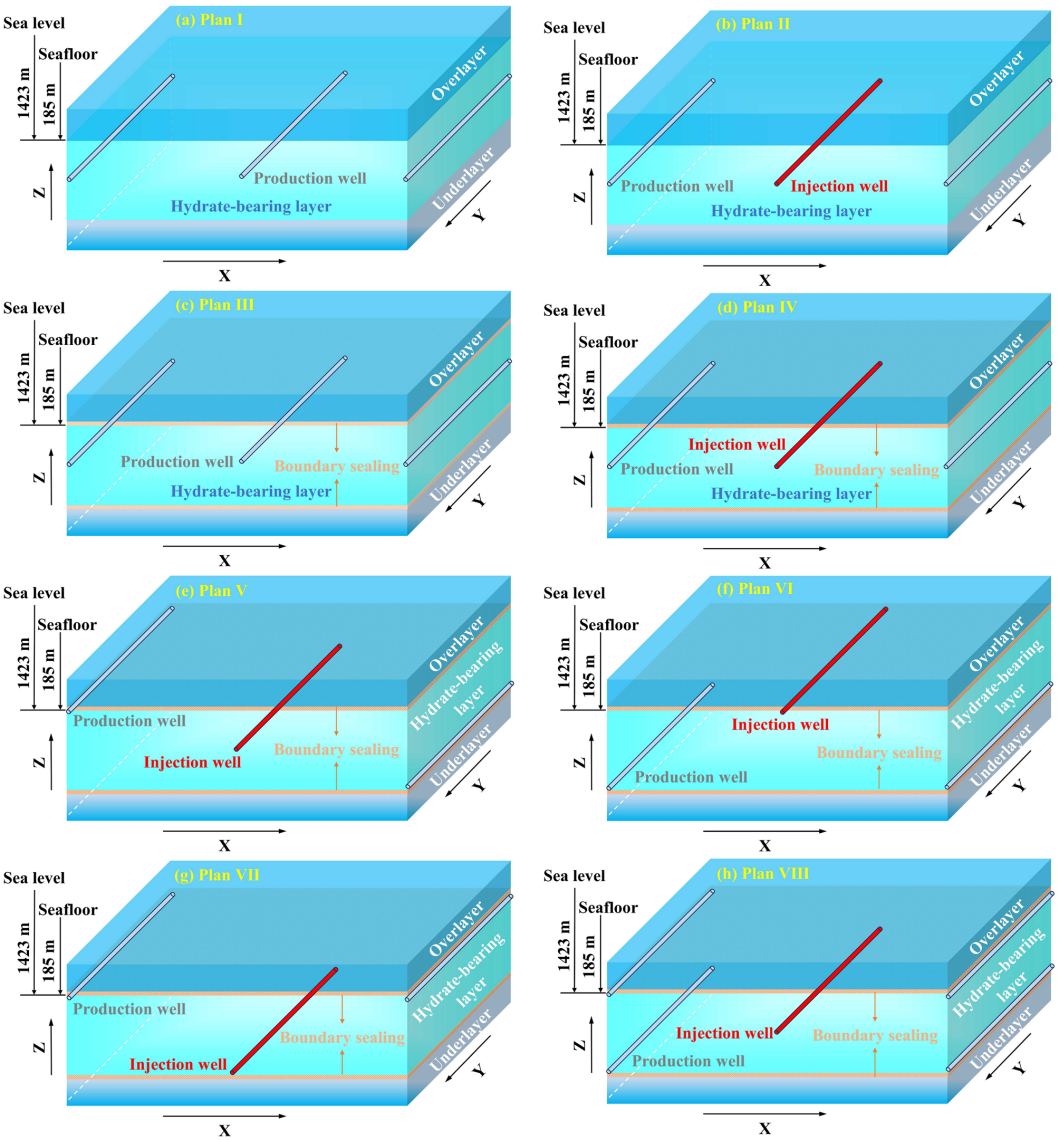
Schematic diagram of development plans.

Horizontal well patterns were used here to obtain considerable drainage area. In addition, due to the limited thickness of the hydrate-bearing layer (HBL), arranging one or two wells vertically can fulfill the NGH decomposition requirements. Consequently, the mainstream horizontal well patterns including three-spot and five-spot were adopted^22,39,40^, as shown in Fig. 1.

In plan I (Fig. 1a), a three-spot well pattern was used, wherein all horizontal wells were located in the middle of the hydrate-bearing layer (HBL) and producing at a constant bottom-hole pressure of 4.5 MPa, which was consistent with field trial production condition^4^.

In plan II (Fig. 1b), the difference from plan I is that the middle horizontal well was used for hot water injection, to evaluate the thermal stimulation potential.

In plans III and IV (Fig. 1c,d), low-permeability sealing zones were created at the boundaries by using sealing and plugging materials. The thickness of the sealed boundaries was assumed to be 1 m, and the permeability was set to 0.0001–1 mD to analyze the stimulation effect of boundary sealing.

In plans V–VIII (Fig. 1e–h), various well patterns were designed, including three-spot with middle injection and up-down production (Fig. 1e), up injection and down production (Fig. 1f), down injection and up production (Fig. 1g), as well as five-spot with central injection and surrounding production (Fig. 1h), to obtain the ideal well pattern.

The specific simulation scenarios are listed in Table 1.

**Table 1:** Simulation scenarios.

Scene No	Plan	k_b_, mD	P_i_, MPa	T_i_, ℃	Scene No	Plan	k_b_, mD	P_i_, MPa	T_i_, ℃
1	I	10	–	–	14	VI	0.0001	20	60
2	II	10	20	60	15	VII	0.0001	20	60
3	III	1	–	–	16	VIII	0.0001	20	60
4	III	0.1	–	–	17	VIII	0.0001	17	60
5	III	0.01	–	–	18	VIII	0.0001	18	60
6	III	0.001	–	–	19	VIII	0.0001	19	60
7	III	0.0001	–	–	20	VIII	0.0001	21	60
8	IV	1	20	60	21	VIII	0.0001	22	60
9	IV	0.1	20	60	22	VIII	0.0001	20	30
10	IV	0.01	20	60	23	VIII	0.0001	20	40
11	IV	0.001	20	60	24	VIII	0.0001	20	50
12	IV	0.0001	20	60	25	VIII	0.0001	20	70
13	V	0.0001	20	60	26	VIII	0.0001	20	80

### Numerical code, initial and boundary conditions

TOUGH + HYDRATE (T+H), developed by Lawrence Berkeley National Laboratory, is a code for the simulation of hydrate geologic systems. It is written in standard FORTRAN 95, and can be run on computing platforms (Macintosh, workstation, PC) for which such compilers are available^41^. Currently, T + H has been widely used in the simulation of various CH_4_-hydrate deposits because of its powerful function in describing methane hydrate phase behavior, heat exchange, and fluid flow in porous media. Moreover, its reliability has been fully examined^42–46^, and thus was employed in this study.

The top and bottom of the model were set as constant temperature–pressure boundaries as sufficient cover thickness was considered. Since the sediments are permeable, it is assumed that pore seawater is connected, allowing good pressure–temperature transformation. As a result, reservoir pressure and temperature can be derived from the hydrostatic pressure gradient and ground temperature gradient, respectively. Other model parameters are given in Table 2.

**Table 2:** Model parameters.

Parameters	Value and unit
Thicknesses of overlayer, HBL, and underlayer	80, 40, and 80 m
Initial average porosity of sediments	0.38
Initial average permeability of sediments	10 mD
Sediment density	2600 kg/m^3^
Dry and wet sediment thermal conductivity	1 and 3.1 W/m/K
Sediment specific heat	1000 J·K/kg
Initial average hydrate saturation	0.40
Gas composition	CH_4_ 100%
Seawater salinity	3.40%
Capillary pressure model^47^	$P_{cap} = - P_{0} \left[ {(S^{*} )^{ - 1/\lambda } - 1} \right]^{1 - \lambda } ,S^{*} = \frac{{\left( {S_{A} - S_{irA} } \right)}}{{\left( {S_{mxA} - S_{irA} } \right)}}$
Maximum aqueous saturation ${\text{S}}_{\text{mxA}}$	1.00
Entry capillary pressure ${\text{P}}_{0}$	1 × 10^5^ Pa
Pore structure index λ	0.45
Reservoir pressure model	$\text{P}={\text{P}}_{\text{atm}}+{\uprho }_{\text{sw}}\cdot \text{g}\cdot \left({\text{D}}_{\text{sw}}+{\text{h}}_{\text{mbsf}}\right)$
Atmospheric pressure ${\text{P}}_{\text{atm}}$	1.013 × 10^5^ Pa
Seawater density ${\uprho }_{\text{sw}}$	1024 kg/m^3^
Gravitational acceleration $\text{g}$	9.80 m/s^2^
Seawater depth ${\text{D}}_{\text{sw}}$	1238 m
Meters below the seafloor in the model ${\text{h}}_{\text{mbsf}}$	105–305 m in our model
Reservoir temperature model	$\text{T}={\text{T}}_{\text{sf}}+\Delta \text{T}\cdot {\text{h}}_{\text{mbsf}}$
Seafloor temperature ${\text{T}}_{\text{sf}}$	4.5 ℃
Ground temperature gradient $\Delta \text{T}$	0.047 ℃/m
Relative permeability model^48^: where ${\text{k}}_{\text{rA}}$ and ${\text{k}}_{\text{rG}}$ are the relative permeabilities of the aqueous and gaseous phases, respectively	$k_{rA} = \left( {\frac{{S_{A} - S_{irA} }}{{1 - S_{irA} }}} \right)^{n} ,k_{rG} = \left( {\frac{{S_{G} - S_{irG} }}{{1 - S_{irA} }}} \right)^{{n_{G} }}$
Permeability reduction exponent $\text{n}$	3.50
Gas permeability reduction exponent ${\text{n}}_{\text{G}}$	3.50
Irreducible aqueous saturation ${\text{S}}_{{\text{ir}}{\text{A}}}$	0.30
Irreducible gas saturation ${\text{S}}_{{\text{ir}}{\text{G}}}$	0.03

## Result analysis

### Stimulation by boundary sealing

**Figure 2:**
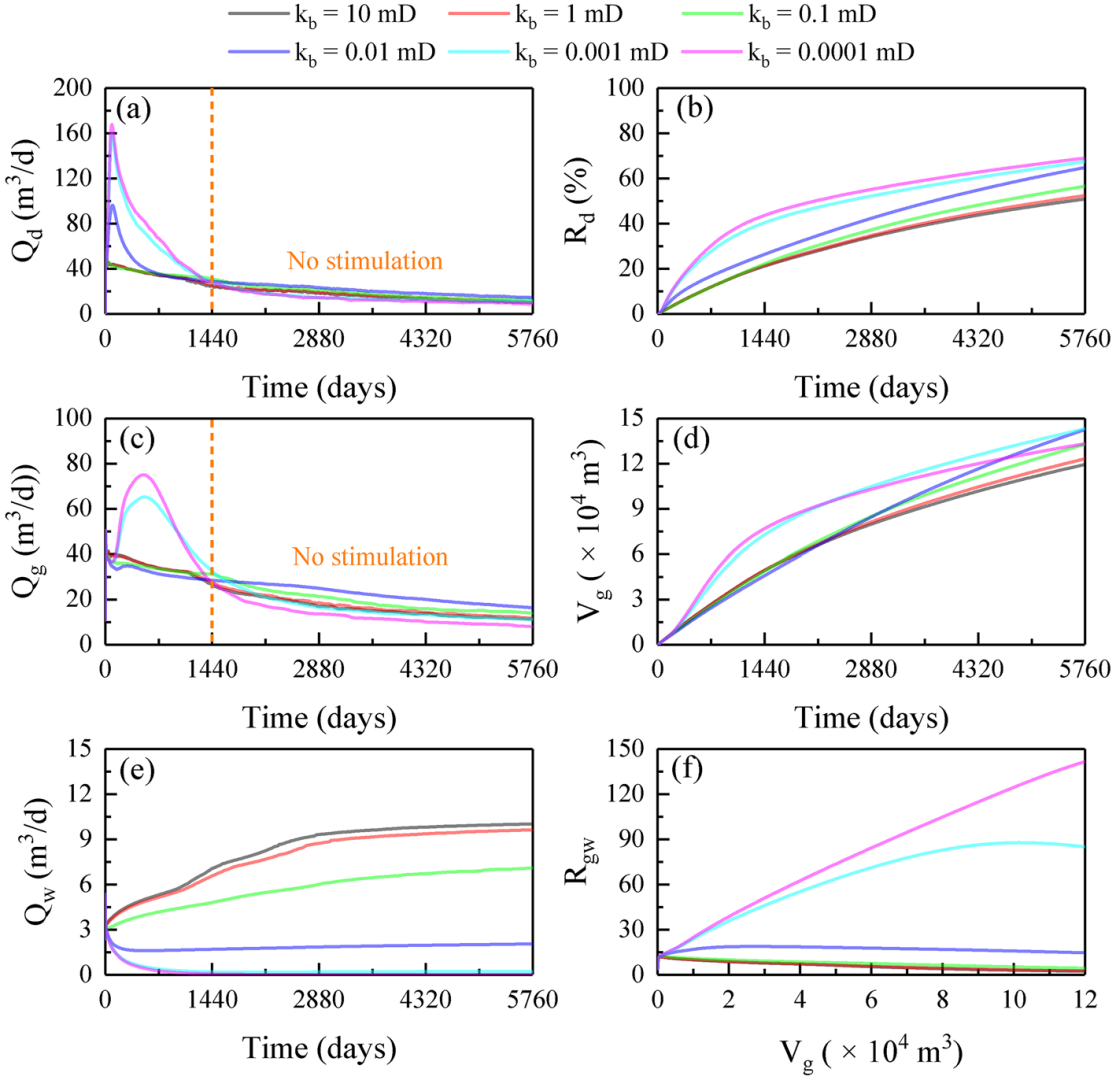
Production dynamics of depressurization at various k_b_ values: (**a**) gas release rate Q_d_; (**b**) hydrate decomposition rate R_d_; (**c**) gas production rate Q_g_; (**d**) cumulative gas production V_g_; (**e**) water production rate Q_w_; (**f**) gas-to-water ratio R_gw_.

**Figure 3:**
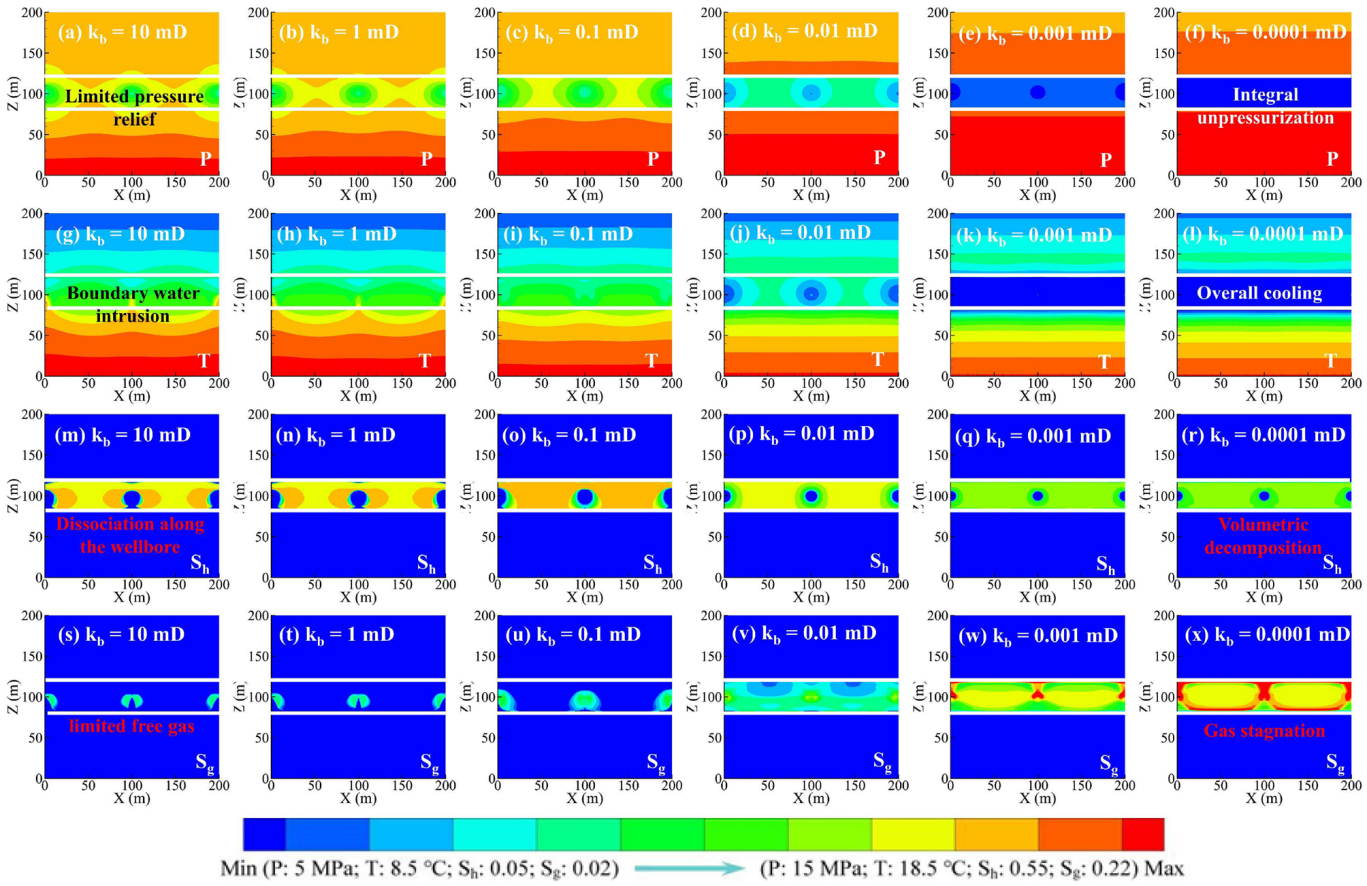
Reservoir physical field distributions for 1440 days of depressurization: (**a–f**) pressure P; (**g–i**) temperature T; (**m–r**) hydrate saturation S_h_; and (**s–x**) gas saturation S_g_.

Figure 2 shows the production dynamics of depressurization at various k_b_ values. The gas release rate from hydrate decomposition (Q_d_) decreased gradually at k_b_ of 0.1–10 mD, whereas at k_b_ of 0.0001–0.01 mD, it showed a rapid rise, followed by (~ 150 days) a sharp decline and then (~ 1440 days) a slow decline (Fig. 2a). The maximum Q_d_ increased from 46 to 168 m^3^/d as k_b_ decreased from 10 to 0.0001 mD, indicating that reducing k_b_ was conducive to hydrate decomposition. This mechanism can be revealed by the reservoir physical field distribution in Fig. 3. As can be seen, the low-pressure zone and the magnitude of the pressure drop in the HBL increased with decreasing k_b_ (Fig. 3a–f), especially when k_b_ reduced to 0.01 mD, the whole HBL was effectively depressurized, resulting in the hydrate dissociation along the borehole changed to volumetric dissociation (Fig. 3g–x). The stimulation effect of boundary sealing was mainly effective in the first 1440 days. After that, the temperature of the HBL decreased significantly due to the heat absorption of hydrate dissociation (Fig. 3g–l), and thus, hydrate decomposition was inhibited by limited reservoir sensible heat. The hydrate decomposition rate (R_d_, defined by the ratio of the mass of the decomposed hydrate to the mass of the original hydrate) over 5760 days increased from 51 to 69% as k_b_ decreased from 10 to 0.0001 mD (Fig. 2b). Therefore, hydrate decomposition is encouraged by sealing boundaries, and reducing k_b_ to less than 0.001 mD is recommended here.

The evolution of the gas production rate (Q_g_) was similar to that of Q_d_ (Fig. 2c). One difference is that there is no initial rise phase of Q_g_ at k_b_ of 0.01 mD due to the retardation of gas production. As k_b_ decreased from 10 to 0.0001 mD, the maximum Q_g_ increased from 50 to 75 m^3^/d. After 1440 days, Q_g_ was the lowest at k_b_ of 0.0001 mD as the whole depressurized HBL cannot provide enough pressure difference for gas flow, resulting in substantial gas hold-up (Fig. 3j–x). The cumulative gas production (V_g_) over 5760 days increased first and then decreased with decreasing k_b_, with a maximum value of 14.25 × 10^4^ m^3^ at k_b_ of 0.01 and 0.001 mD (Fig. 2d). Consequently, there exists a critical k_b_ value for gas enhancement, and 0.01–0.001 mD is suggested.

The water production rate (Q_w_) increased rapidly and then (~ 2880 days) slowly at k_b_ of 0.1–10 mD, whereas it decreased sharply and then stabilized at k_b_ of 0.0001–0.01 mD (Fig. 2e). The sources of produced water include free water in the HBL, hydrate dissociation water, and boundary water in the boundary layers. It can be concluded that boundary water intrusion was inhibited by decreasing k_b_ and thus reducing water yield. The gas-to-water ratio (R_gw_, defined by the ratio of cumulative gas production to total water production) decreased at k_b_ values of 0.1–10 mD, with a maximum value lower than 15; whereas increased linearly at k_b_ of 0.0001 mD, finally reaching a value of 142 (Fig. 2f). Consequently, boundary sealing is effective for water control.

### Stimulation by hot water injection and boundary sealing

**Figure 4:**
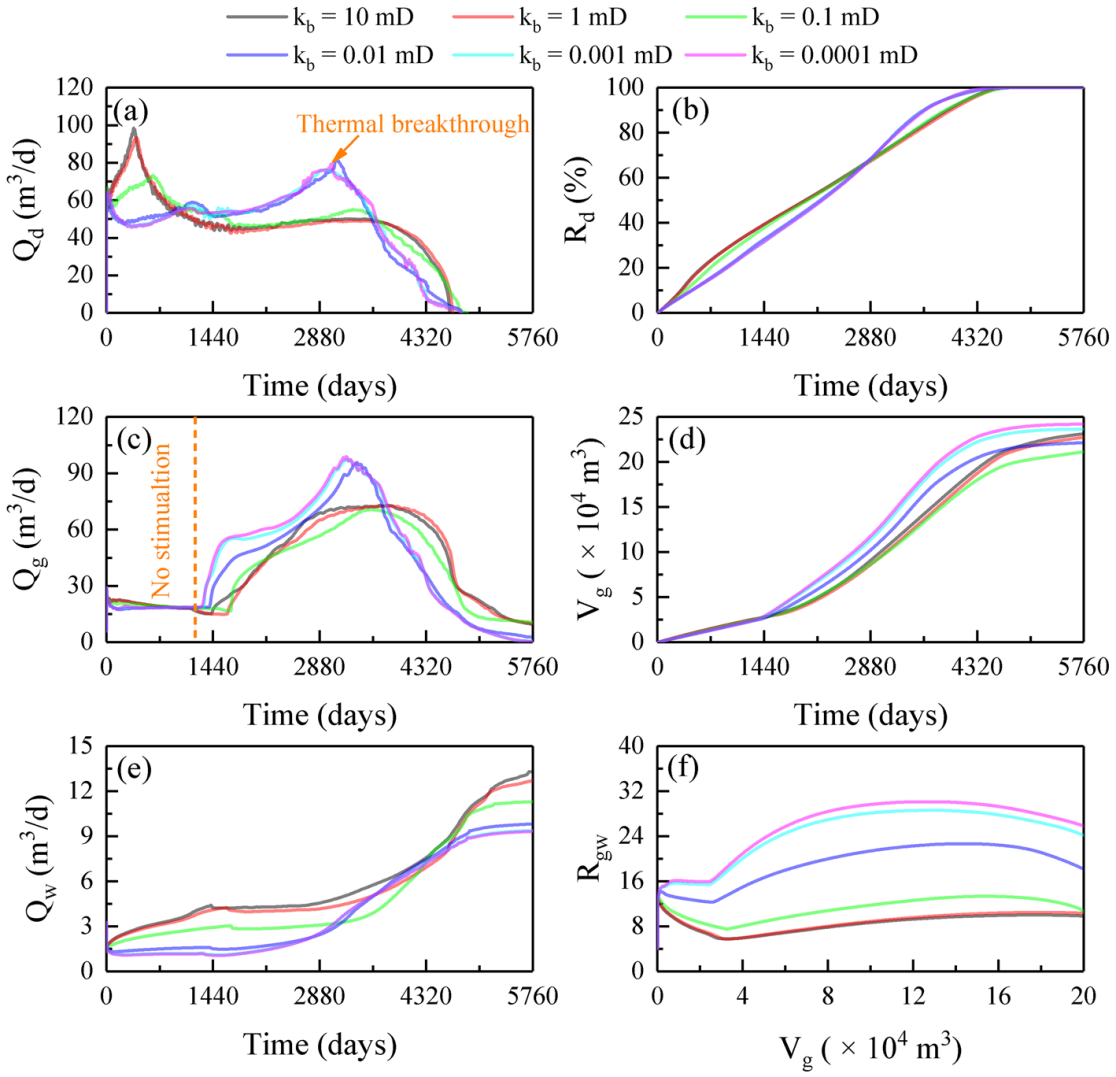
Production dynamics of injection-production scenarios at various k_b_ values: (**a**) Q_d_; (**b**) R_d_; (**c**) Q_g_; (**d**) V_g_; (**e**) Q_w_; (**f**) R_gw_.

**Figure 5:**
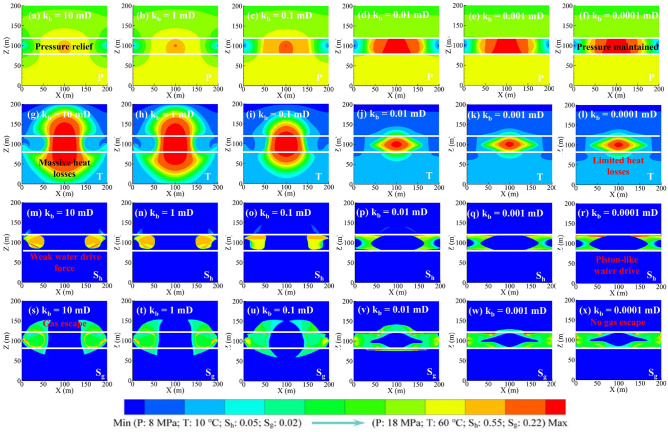
Reservoir physical field distributions for 2880 days of depressurization and hot water injection at various k_b_ values: (**a–f**) P; (**g–i**) T; (**m–r**) S_h_; (**s–x**) S_g_.

Figure 4 exhibits the production dynamics of injection-production scenarios at various k_b_ values. As can be seen, the evolution of Q_d_ at k_b_ of 0.1–10 mD presented four phases (Fig. 4a): first, with the dissociation of hydrate around the injection well, the hot water spread area increased gradually, leading to a gradual increase in Q_d_; when the thermal decomposition front extended to the boundary layers, the high injection pressure was released (Fig. 5a–c); then entered the second phase (~ 360 days), and Q_d_ gradually decreased due to a substantial amount of hot water transport to the boundary layers (Fig. 5g–i); in the third phase (~ 800 days), Q_d_ was stabilized at about 40 m^3^/d with stable thermal stimulation; once the hot water broke through to production wells, (a phenomenon known as water flooding in water-driven reservoirs and corresponding to the breakthrough time T_bt_), it entered into the fourth phase (~ 4000 days), and Q_d_ decreased dramatically as hypertonic channels were formed (significant increase in effective reservoir permeability after hydrate between injection and production wells was decomposed). The difference is that when k_b_ was reduced to 0.01 mD, Q_d_ decreased initially as the stagnant gas inhibited the hot water injection. Subsequently (~ 200 days), Q_d_ increased with the injection of hot water. Interestingly, when the thermal decomposition front reached the boundary layers, the high injection pressure (Fig. 5d–f) was maintained and the hot water loss was avoided (Fig. 5j–l), and therefore, Q_d_ continued to increase after a slight fluctuation until it reached the peak value (80 m^3^/d). After that (~ 3000 days), Q_d_ decayed sharply after T_bt_. Overall, the influence of k_b_ on the final R_d_ was limited (Fig. 5m–r) because hydrate decomposition cycles (T_hd_) were all approximately 3500 days (Fig. 4b). Thus, boundary sealing has a significant influence on hydrate decomposition behavior in the injection-production scenarios but hardly affects T_hd_ because it mainly controlled by the thermal breakthrough.

The evolution of Q_g_ at different k_b_ values was similar, as shown in Fig. 4c. Initially, Q_g_ was stabilized at 18 m^3^/d and was contributed by depressurization. Subsequently, thermal decomposition gas was driven to the production wells (~ 1440 days), and thus Q_g_ increased sharply. The maximum Q_g_ at k_b_ of 0.0001–0.01 mD was higher than 95 m^3^/d, while it was lower than 75 m^3^/d at k_b_ of 0.1–10 mD. The mechanism for this is twofold: first, the high injection pressure (Fig. 5a–f) improved the water driving force; and second, hot water loss (Fig. 5g–l) and gas escape (Fig. 5s–x) was avoided. The final V_g_ initially increased and then decreased with reducing k_b_, with a maximum value of 24.20 × 10^4^ m^3^ and a gas recovery period (T_gr_) of 5600 days at k_b_ of 0.0001 mD (Fig. 4d). Thus, hot water loss and gas escape were addressed by reducing k_b_ to 0.0001 mD.

With T_bt_ as the node (~ 2880 days), the change in Q_w_ can be divided into low- and high-yield phases afterward, as shown in Fig. 4e. For R_gw_, it first decreased and then increased, with a low level lower than 15 at k_b_ of 0.1–10 mD; however, when k_b_ reduced to 0.01 mD, it increased after the thermal decomposition gas was produced, and decreased after T_bt_ (Fig. 4f). The final R_gw_ increased from 10 to 26 as k_b_ reduced from 10 to 0.0001 mD. As a result, boundary sealing is still effective for water control in injection-production scenarios.

### Influence of well patterns

**Figure 6:**
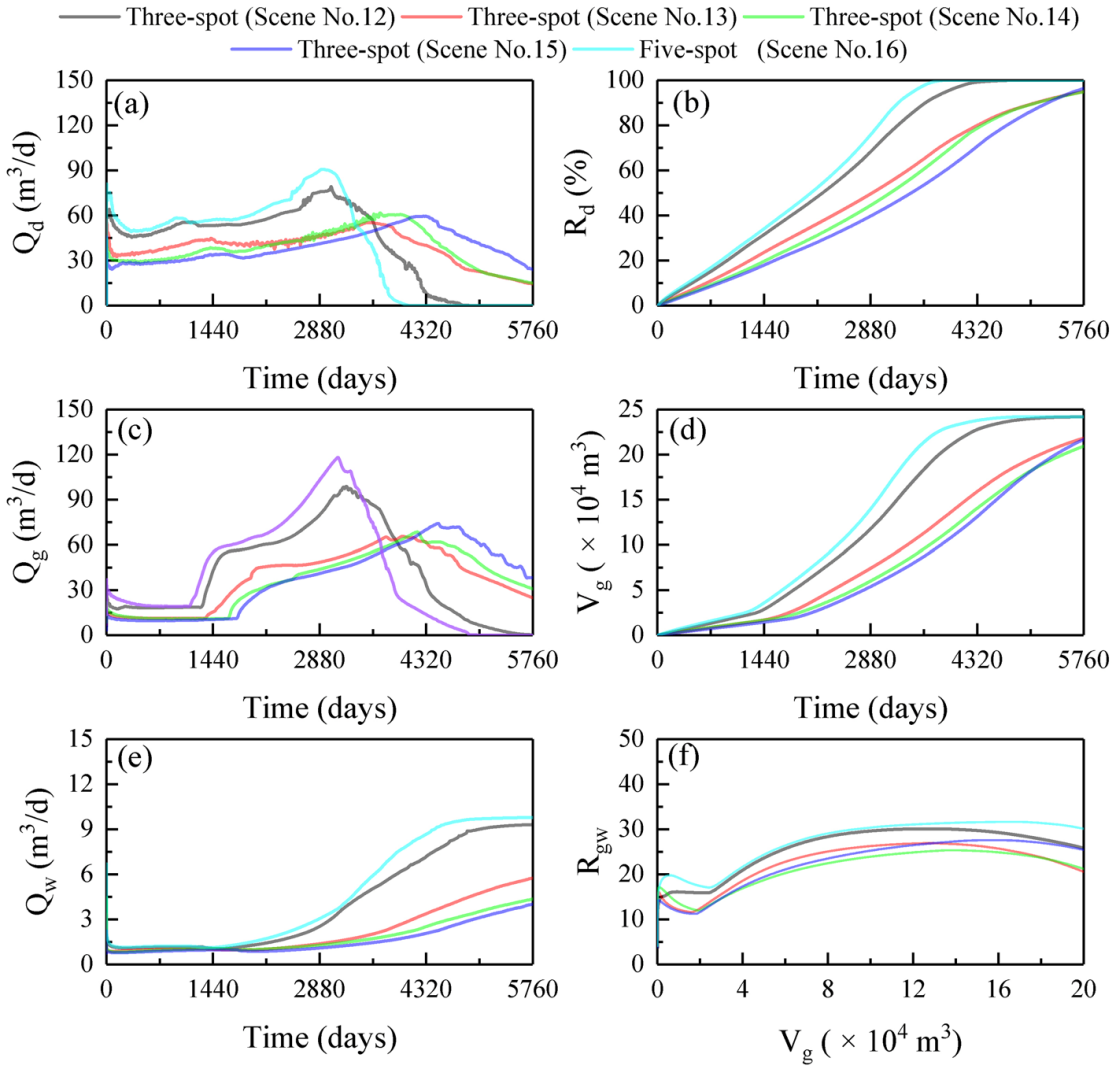
Production dynamics under various injection-production well patterns: (**a**) Q_d_; (**b**) R_d_; (**c**) Q_g_; (**d**) V_g_; (**e**) Q_w_; (**f**) R_gw_.

Figure 6 shows the production dynamics under various injection-production well patterns. In the three-spot well patterns, Q_d_, Q_g_, and Q_w_ were the higher in Scene No.12, and T_bt_ was also the shortest (Fig. 6a,c,e). The mechanism is that the pressure gradient between the injection and production wells is greater under a smaller well spacing, which is more favorable for hot water penetration, hydrate decomposition, as well as gas and water recovery. Compared with the three-spot, the five-spot was more conducive to pressure propagation and hot water penetration, which further promotes hydrate decomposition and gas recovery. The T_hd_ and T_gr_ of the five-spot were 3878 and 4890 days, shorting at least 903 and 680 days, respectively, compared to the three-spot (Fig. 6a–d). The final R_gw_ of the five-spot was 30, at least 5 higher than those of the three-spots (Fig. 6f). Therefore, the five-spot well pattern is the most ideal in the current simulations.

### Influence of injection pressure

**Figure 7:**
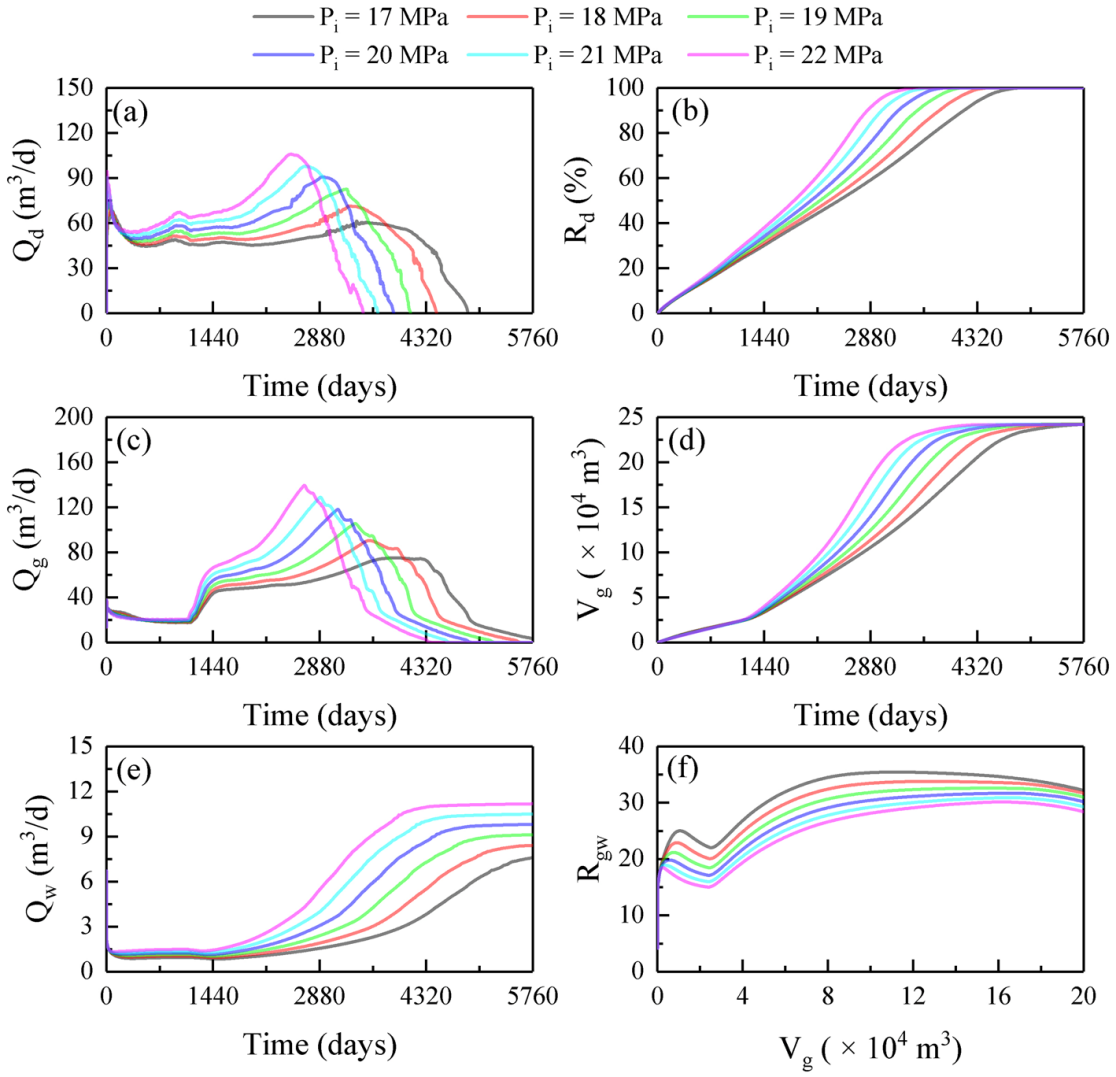
Production dynamics at various P_i_ values: (**a**) Q_d_; (**b**) R_d_; (**c**) Q_g_; (**d**) V_g_; (**e**) Q_w_; (**f**) R_gw_.

Figure 7 shows the production dynamics at various P_i_ values. As can be seen, when P_i_ increased from 17 to 22 MPa, the maximum Q_d_ increased from 60 to 116 m^3^/d, T_bt_ decreased from 3750 to 2640 days, and T_hd_ shortened from 4870 to 3460 days (Fig. 7a,b); the maximum Q_g_ increased from 75 to 140 m^3^/d, and T_gr_ decreased from 5940 to 4340 days (Fig. 7c,d); the maximum Q_w_ increased from 7.5 to 11, and the final R_gw_ decreased from 32 to 28 (Fig. 7e,f). The stimulation mechanism is that improving P_i_ increases the hot water injection rate and water-driven force, thereby favoring reservoir heating and gas–water recovery. Consequently, increasing P_i_ could enhance the extraction efficiency, but simultaneously induce high water production.

### Influence of hot water temperature

**Figure 8:**
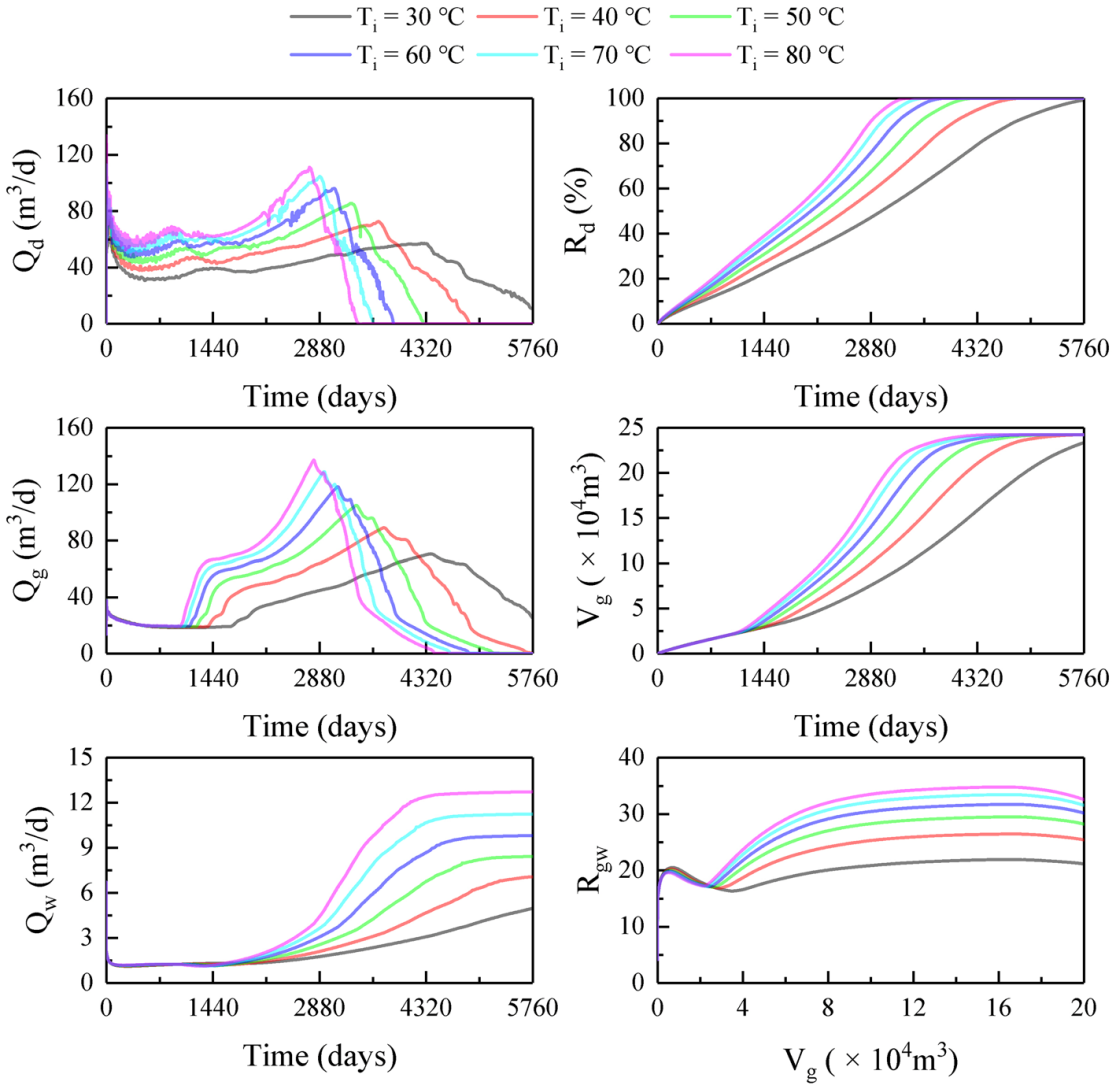
Production dynamics at various T_i_ values: (**a**) Q_d_; (**b**) R_d_; (**c**) Q_g_; (**d**) V_g_; (**e**) Q_w_; (**f**) R_gw_.

Figure 8 shows the production dynamics at various T_i_ values. Similar to increasing P_i_, increasing T_i_ was also conducive to pressure propagation, reservoir heating, hydrate dissociation, and gas–water recovery. Specifically, when T_i_ increased from 30 to 80 ℃, the maximum Q_d_ increased from 59 to 120 m^3^/d, T_bt_ decreased from 4215 to 2782 days (Fig. 8a), and T_hd_ shortened from 6036 to 3382 days (Fig. 8b); the maximum Q_g_ increased from 70 to 137 m^3^/d (Fig. 8c); T_gr_ decreased from 6811 to 4415 days (Fig. 8d); and the maximum Q_w_ increased from 4.9 to 12 (Fig. 8e). Different from increasing P_i_, increasing T_i_ provided a more significant enhancement of gas production, resulting in an increase in the final R_gw_ from 21 to 33 as T_i_ increased from 30 to 80 ℃ (Fig. 8f). The mechanism is that water injection is not significantly strengthened due to the constant P_i_. Therefore, increasing T_i_ helps to control water while improving extraction efficiency.

## Discussion

**Figure 9:**
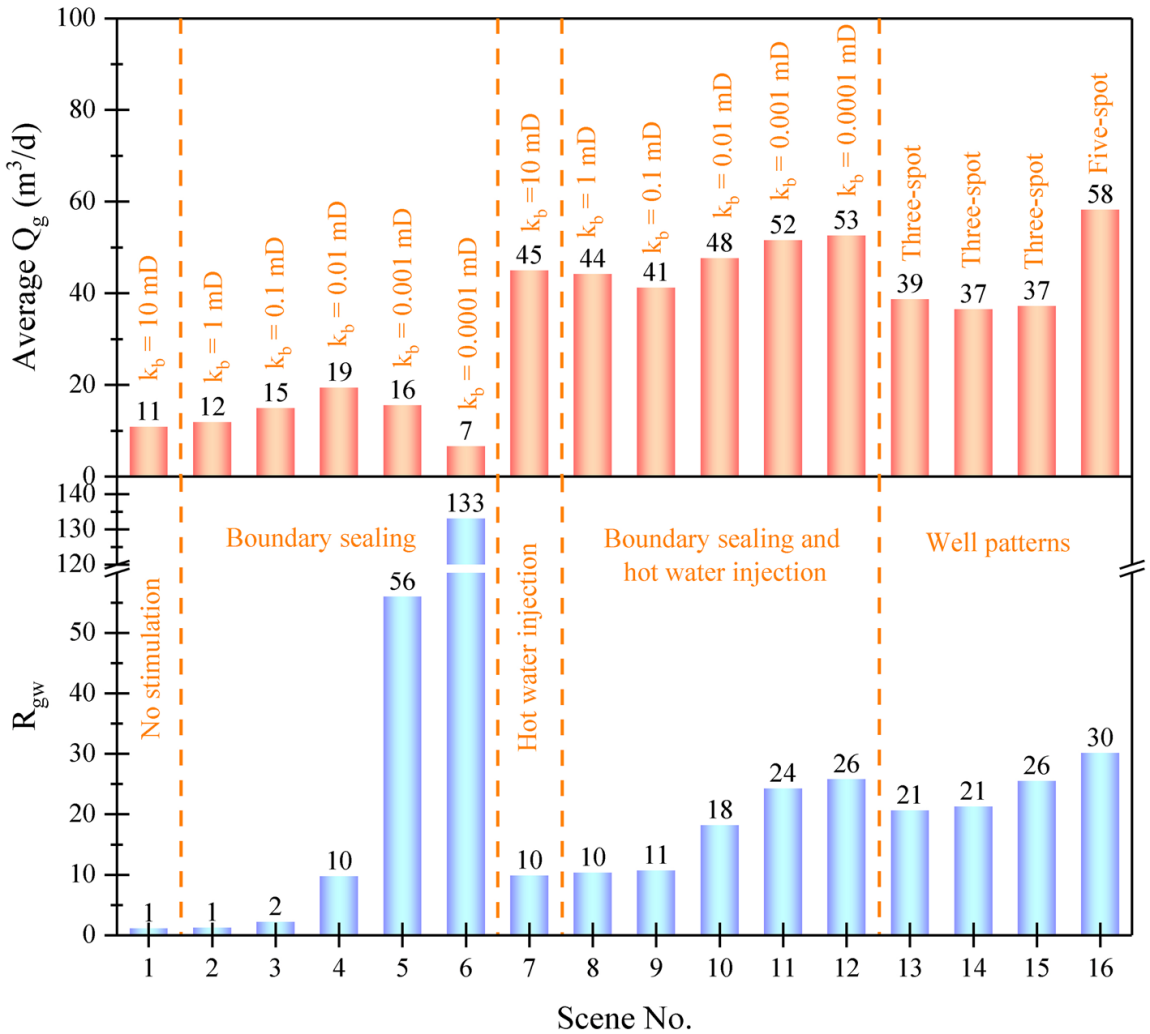
Average Q_g_ and R_gw_ at V_g_ of 20 × 10^4^ m^3^ in Scene Nos. 1–16.

From the analysis of the above results, it is found that boundary sealing prevented boundary water intrusion, and the hydrate volumetric decomposition was realized by the effective depressurization of the whole reservoir. However, the limited environment sensible heat was insufficient for sustained hydrate decomposition, and a substantial amount of gas was retained in the reservoir due to the lack of gas driving force. Hot water injection alone can provide adequate heat for hydrate decomposition. However, when the hot water breaks through to the open boundaries, the injected hot water and free gas will leak into the boundary layers under the drive of high injection pressure. This is detrimental to energy utilization and poses a serious environmental safety risk. The combination of boundary sealing and hot water injection realized a piston-like water-driven gas extraction mode, addressing the issues of insufficient reservoir sensible heat, water intrusion, as well as gas retention and escape. Here, the synergistic effect on gas enhancement and water control is analyzed based on average Q_g_ and R_gw_ at V_g_ of 20 × 10^4^ m^3^, as presented in Fig. 9. When only boundary sealing is applied, average Q_g_ first increases and then decreases with a maximum value of 19 m^3^/d at k_b_ of 0.1 mD, and R_gw_ increases from 1 to 133 as k_b_ decreases from 10 to 0.0001 mD, indicating that boundary sealing is effective for water control but not always favorable for gas recovery, thereby exhibiting a low-gas and low-water production pattern; when only hot water injection is used, average Q_g_ increases from 11 to 45 m^3^/d, and R_gw_ increases from 1 to 10. This indicates that the gas enhancement is more significant than the increase of water yield, presenting a high-gas and high-water production pattern; when both boundary sealing and hot water injection are adopted, the average Q_g_ increases first and then decreases with a maximum value of 53 m^3^/d at k_b_ of 0.0001 mD, and R_gw_ increases from 10 to 26 as k_b_ decreased from 10 to 0.0001 mD, suggesting that sealed boundaries further improve the thermal stimulation potential while reducing water yield, thereby resulting a high-gas and low-water production pattern. The values of average Q_g_ in Scene Nos. 6 and 7 are 7 and 45 m^3^/d, their sum (52 m^3^/d) is lower than that of Scene No. 12 (53 m^3^/d). Consequently, hot water injection and boundary sealing play a synergistic effect like “1 + 1 ≥ 2” on gas enhancement.

In addition, for three-spot well patterns, average Q_g_ and final R_gw_ are the highest (53 m^3^/d and 26) when the injection and production wells are located in the middle of the HBL. However, they were lower than those (58 m^3^/d and 30) of the five-spot, indicating the five-spot was better. Considering the multi-well synergistic effect^25^, increasing the complexity of the well pattern such as seven-spot and nine-spot may further improve the extraction efficiency, but simultaneously increase the drilling cost. In particular, the influence of well location on the multi-phase fluid flow is more complex, and therefore, further systematic investigation is needed.

**Figure 10:**
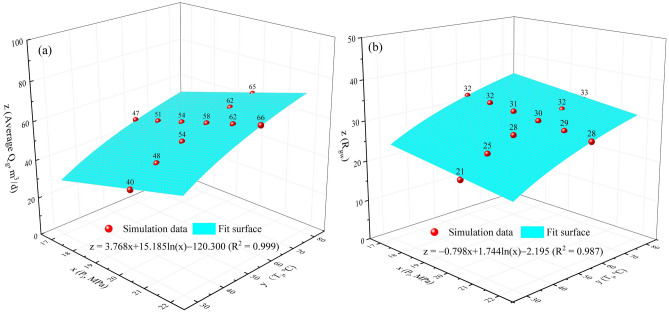
Relationships of P_i_ and T_i_ with (**a**) average Q_g_ and (**b**) R_gw_ at V_g_ of 20 × 10^4^ m^3^.

To investigate the influence rule of injection parameters on gas–water production, the relationships of P_i_ and T_i_ with average Q_g_ and R_gw_ at V_g_ of 20 × 10^4^ m^3^ are fitted, respectively, as presented in Fig. 10. As can be seen, when P_i_ increases from 17 to 22 MPa, average Q_g_ increases linearly from 47 to 66 m^3^/d, whereas R_gw_ decreases linearly from 32 to 29, indicating that water yield was enhanced more significantly_._ Both average Q_g_ and R_gw_ increase logarithmically with increasing T_i_ from 30 to 80 ℃, from 40 to 65 m^3^/d, and from 21 to 33, respectively, indicating that gas yield is enhanced more significantly. As a result, high-pressure and high-temperature injection is conducive to gas recovery, while low-pressure and high-temperature injection favors water control. However, overall, compared to the enhancement of P_i_ on the average Q_g_, its decrease in R_gw_ is very limited, with fitting coefficients of 3.768 and 0.798, respectively. Consequently, high-pressure and high-temperature injection is recommended for gas enhancement and water control.

## Conclusions

This research proposes a novel reservoir stimulation method combining boundary sealing and hot water injection for challenging hydrates, and the stimulation effect was numerically evaluated. The following conclusion can be drawn.

1. Boundary water intrusion is effectively addressed by sealing open boundaries, thus promoting pressure drop propagation and realizing the volumetric decomposition of hydrates. However, its stimulation validity is limited (~ 3 years), after which a substantial amount of decomposed gas is trapped in the reservoir due to insufficient formation energy and environmental heat, resulting in productivity not always increasing with the decrease of boundary permeability.
2. Hot water loss and gas escape are avoided by decreasing the boundary permeability to 0.0001 mD. Furthermore, a piston-like water drive similar to secondary recovery is realized, thereby drastically improving the extraction efficiency. Furthermore, compared with the three-spot well patterns, the five-point is more conducive to gas enhancement and water control.
3. The enhancement of water production by increasing injection pressure is more significant, leading to a slight increase in the gas–water ratio; whereas the enhancement of gas production by increasing hot water temperature is more significant, resulting in a decrease in the gas–water ratio. As a whole, high-temperature and high-pressure injection is recommended.
4. This study offers a novel promising development plan for marine challenging hydrates. However, it is important to point out that to obtain universal rules, the current simulations were performed under relatively ideal geoengineering conditions, considering only a single HBL and three-spot and five-spot well patterns. Given the heterogeneity of hydrate reservoir, the effectiveness of boundary sealing, and other unconsidered complex well patterns such as seven-spot and nine-spot potentially affect mass and heat transfer behavior, further research in development plan optimization is needed, with a comprehensive consideration of these issues.

## Data Availability

Data used in this study are available from the corresponding author by request.
